# Arsenic in brown rice: do the benefits outweigh the risks?

**DOI:** 10.3389/fnut.2023.1209574

**Published:** 2023-07-14

**Authors:** Lihchyun Joseph Su, Tung-Chin Chiang, Sarah N. O’Connor

**Affiliations:** ^1^Peter O’Donnell Jr. School of Public Health, University of Texas Southwestern Medical Center, Dallas, TX, United States; ^2^Fay W. Boozman College of Public Health, University of Arkansas for Medical Sciences, Little Rock, AR, United States

**Keywords:** arsenic, exposure, brown rice, cancer, obesity, cardiovascular health

## Abstract

Brown rice has been advocated for as a healthier alternative to white rice. However, the concentration of arsenic and other pesticide contaminants is greater in brown rice than in white. The potential health risks and benefits of consuming more brown rice than white rice remain unclear; thus, mainstream nutritional messaging should not advocate for brown rice over white rice. This mini-review aims to summarize the most salient concepts related to dietary arsenic exposure with emphasis on more recent findings and provide consumers with evidence of both risks and benefits of consuming more brown rice than white rice. Despite risk-benefit assessments being a challenging new frontier in nutrition, researchers should pursue an assessment to validate findings and solidify evidence. In the interim, consumers should be cognizant that the dose of arsenic exposure determines its toxicity, and brown rice contains a greater concentration of arsenic than white rice.

## Introduction

In recent years, gluten-free, dairy-free, and plant-based dieting has increased in popularity, and rice is a common substitute. The potential health risks and benefits of consuming more brown rice than white rice remain unclear. Despite this, brown rice is often advocated as a healthier alternative to white rice in mainstream diet and nutritional messaging. Evidence of any protective effect of consuming more brown rice than white is limited.

Even though *in-vitro* and animal studies using nutrients and fiber extracted from brown rice have demonstrated improved cardiovascular function and prevention of heart diseases (1–5), these studies fail to utilize the whole grain of brown rice. Additionally, Sun et al. (6) found that when compared to white rice intake, brown rice intake reduced the risk of type 2 diabetes by 16%. The study, however, lacked diversity and included a homogenous population of European descent health professionals.

Current literature widely includes animal studies and primarily examines the benefits of consuming whole grains, not specifically brown rice. Other study limitations fail to provide solid evidence for the health benefits of consuming brown rice over white rice. There is a clear lack of research focusing on human consumption of brown rice that includes a risk-benefit approach. Risk-benefit assessment of foods is a challenging new frontier in food safety research. The assessment estimates human health benefits and risks following exposure (or lack thereof) to a particular food or food component and integrates them into comparable measures (7–9).

What might continue diminishing the evidence between brown rice and its human health benefits? Brown rice contains a greater arsenic concentration than white rice, and the human health risks associated with dietary arsenic exposure are well-established.

### Dietary exposure to arsenic

Arsenic is ubiquitous in the environment and is a global public health concern. Arsenic is a well-known carcinogenic, mutagenic, and toxic environmental element that occurs as inorganic arsenic and organoarsenical compounds (10). Arsenic can be found in food, water, soil, and airborne particles. Inorganic forms of arsenic are found in the environment dissolved in water, and human exposure occurs through drinking water. Additionally, diet is an alternative source of exposure through the consumption of plant-based foods such as wheat, rice, and vegetables grown in contaminated soil and animal products such as dairy, milk, and fish exposed to contaminated feed. Even soil from organic farms can have remnants of arsenic due to historical pesticide use.

Because of the rapid globalization in the food trade, the ingestion of arsenic through rice consumption is not limited to a regional issue but a worldwide health concern (11, 12). A study of more than 204 rice samples sold in the U.S. found that rice grown in certain Southern states, which accounts for more than 47% of the U.S. market, had the highest arsenic content compared to the rice imported from Asia or grown in California (13). A 2017 study estimated Americans’ inorganic arsenic exposures from drinking water and rice. It concluded that rice consumption might account for as much inorganic arsenic exposure as drinking water in some U.S. populations (14).

The U.S. Environmental Protection Agency set a limit for total arsenic in drinking water at 10 parts per billion (ppb). However, no such limit exists for food or other beverages. Thus, rice can contain levels of inorganic arsenic that surpass the limit set for arsenic in drinking water. In 2014, a Consumer Reports analysis of the U.S. Food and Drug Administration (FDA) data on 656 rice products confirmed the worrisome levels of arsenic exposure from white and brown rice. In rice, inorganic arsenic is found in the two outer layers of the grain (i.e., bran and germ), and the bran and germ are removed to refine the grain into white rice. Thus, a greater concentration of arsenic is found in brown rice than in white rice. In the previously cited Consumer Reports study, brown rice contained 80 percent more inorganic arsenic on average than white rice of the same type (15).

In response to concerns raised by the public, the FDA Center for Food Safety and Applied Nutrition conducted an assessment based on the existing evidence of health risks from inorganic arsenic in rice and products that contain rice (16). The investigation concluded that the average concentrations of inorganic arsenic are 92 ppb in white rice, 154 ppb in brown rice, 104 ppb in infants’ dry white rice cereal, and 119 ppb in infants’ dry-brown rice cereal. The data demonstrated that inorganic arsenic concentration is 1.5 times higher in brown rice than in white rice. The expert panel concluded that the risk of exposure and associated health condition(s) increases proportionally with consumption and depends on the type of rice consumed. Notably, the FDA assessment focused on lung and bladder cancer. The expert panel concluded that cancer cases would have increased by 148.6% if rice consumption increased from less than one serving per day, the current level, to precisely one serving per day (16). Although none of the products analyzed in the Consumer Reports study reached the acute toxicity level, the health effects of long-term low-dose exposure are unclear. According to the U.S. FDA, the adverse health effects of arsenic exposure depend on various factors, such as the type of arsenic (organic or inorganic), the level of exposure, and the age of the person exposed to the arsenic. Many studies have linked arsenic exposure to cancers, cardiovascular disease, diabetes mellitus, hypertension, and obesity (17, 18).

### Arsenic exposure and disease

Various studies have demonstrated that pregnant females, fetuses, and neonates suffer adverse pregnancy outcomes when exposed to arsenic (19–23). *In utero*, inorganic arsenic exposure was positively associated with DNA damage in offspring (24). Recent health risk assessments reported that the consumption of arsenic-containing rice and rice-based foods (e.g., cereals, cakes, and crackers) led to increased cancer risks, especially in subpopulations of infants and children (25–29).

In humans, inorganic arsenic compounds are converted to trivalent arsenic (As^III^) and pentavalent arsenate (As^V^). As^V^ is rapidly converted to As^III^. As^III^ species are more toxic and bioactive than As^V^ species, both because of the greater chemical reactivity of As^III^ and because As^III^ enters cells more easily (30). Both arsenic species coexist in drinking water with varying toxicity (31). According to the International Agency for Research on Cancer (IARC), there is sufficient evidence in humans for the carcinogenicity of mixed exposure to inorganic arsenic compounds, including arsenic trioxide, arsenite, and arsenate. Exposure to arsenic stimulates epigenetic disruption in various cellular processes, which can cause cancer. Presently, three modes (i.e., chromosomal abnormality, oxidative stress, and altered growth factors) of arsenic carcinogenesis have a degree of positive evidence, both in experimental systems (animal and human cells) and in human tissues (32–34). The IARC concludes that different inorganic arsenic species should be considered carcinogenic independent of the mechanisms of carcinogenic action and independent of which metabolites are the ultimate carcinogen (30).

Chronic arsenic exposure through consuming certain foods and contaminated water has been associated with an increased risk of prostate, lung, bladder, pancreatic, and skin cancer (31, 35–40). According to the U.S. National Cancer Institute, cancers of the digestive tract, liver, kidney, and lymphatic and hematopoietic systems have also been linked to arsenic exposure. Additionally, arsenic trioxide (As_2_O_3_) treatment in human fibroblasts was shown to disrupt the normal function of the DNA repair pathway and increase genomic instability (24), such that women carrying specific BRCA-1 mutations (e.g., 5382insC, C61G, and 4153delA) were found to be at higher risk of breast cancer with increased arsenic exposure (41).

Arsenic is one of many environmental pollutants linked to metabolic syndrome development (17, 18, 42). Metabolic risk factors that lead to a diagnosis of arsenic-induced metabolic syndrome include having a large waistline, high blood pressure, elevated fasting blood sugar, high triglyceride level, and low HDL cholesterol. Those risk factors are identical to the ones for cardiovascular diseases.

Epidemiological studies have shown that the cardiovascular system is susceptible to long-term ingestion of arsenic (43). Noticeable effects include hypertension and increased cardiovascular disease mortality (43). A growing body of literature suggests that DNA methylation, one of the most frequently researched epigenetic mechanisms, is associated with various outcomes in response to exposure to heavy metals like arsenic (44–47). A recent study proposed epigenetic dysregulation as a critical arsenic-related cardiovascular disease (CVD) mechanism. Researchers conducted a mediation analysis to assess the potential role of DNA methylation on arsenic-related CVD. Results supported that blood DNA methylation influences arsenic-related CVD, and the results were replicated in a mouse model and three independent and diverse human cohorts (48).

In addition, arsenic exposure is also suspected to be related to obesity (24, 49). We recently found a significant dose–response relationship between arsenic concentration and obesity among 270 postmenopausal women randomly selected from a study cohort where most rice is produced (50). Furthermore, we also found a significantly positive association between weight gain velocity and salivary arsenic concentration in the same study. The mechanism for arsenic exposure and obesity is unclear. Arsenic upregulates the cytokine IL-6 expression in various cell types (51). IL-6, a pro-inflammatory cytokine and an anti-inflammatory myokine, is hypothesized to increase the amount of free fatty acids in the body, thus increasing obesity. Another explanation involves regulating glucose uptake by the glucose transporter 4 protein (GLUT4) in adipose and skeletal muscles (52). A recent study determined that low-dose exposure to arsenic for 8 weeks decreased GLUT4 expression (53). When GLUT4 is silenced, the glucose is not effectively transported into the cell. Research has further identified that steady-state glucose homeostasis dysregulations are due to arsenic exposure (54). Although the mechanism remained largely unknown, patterns of dyslipidemia influenced by arsenic have been identified (55).

## Conclusion

There is a clear lack of research focusing on human consumption of brown rice that includes a risk-benefit approach. The fact that brown rice contains more arsenic than white rice cannot be denied, and the human health risks associated with dietary arsenic exposure are well-established. Health effects of arsenic exposure depend on various factors, such as the type of arsenic (organic or inorganic), the level of exposure, and the age of the person exposed to the arsenic. Arsenic exposure has been associated with cancers, cardiovascular disease, diabetes mellitus, hypertension, and obesity. Despite risk-benefit assessment of foods being a challenging new frontier in food safety research, future studies should include an assessment to validate findings and solidify evidence. In the interim, consumers should be cognizant that the dose of arsenic exposure determines its toxicity, and brown rice contains a greater concentration of arsenic than white rice.

## Author contributions

LS drafted the manuscript. LS, T-CC, and SO’C reviewed and completed the manuscript. All authors contributed to the article and approved the submitted version.

## Conflict of interest

The authors declare that the research was conducted in the absence of any commercial or financial relationships that could be construed as a potential conflict of interest.

## Publisher’s note

All claims expressed in this article are solely those of the authors and do not necessarily represent those of their affiliated organizations, or those of the publisher, the editors and the reviewers. Any product that may be evaluated in this article, or claim that may be made by its manufacturer, is not guaranteed or endorsed by the publisher.
